# One-step synthesis of magnetic-TiO_2_-nanocomposites with high iron oxide-composing ratio for photocatalysis of rhodamine 6G

**DOI:** 10.1371/journal.pone.0221221

**Published:** 2019-08-19

**Authors:** En Xie, Lei Zheng, Xinyang Li, Yingying Wang, Junfeng Dou, Aizhong Ding, Dayi Zhang

**Affiliations:** 1 College of Water Resources and Civil Engineering, China Agricultural University, Beijing, PR China; 2 College of Water Sciences, Beijing Normal University, Beijing, PR China; 3 Lancaster Environment Centre, Lancaster University, Lancaster, United Kingdom; 4 School of Civil Engineering, Beijing Jiaotong University, Beijing, China; 5 School of Environment, Tsinghua University, Beijing, PR China; Brandeis University, UNITED STATES

## Abstract

In the study, a facile one-step method for synthesizing magnetic-TiO_2_-nanophotocatalysts was developed. With the same composing ratio of 0.5 and 0.35 (Fe:Ti, mole:mole), we prepared two types of magnetic-TiO_2_-nanocomposites as one-step synthesized Fe_x_O_y_-composed TiO_2_ (Fe_x_O_y_/TiO_2_-0.5 and Fe_x_O_y_/TiO_2_-0.35) and two-step synthesized core-shell Fe_x_O_y_@TiO_2_ (Fe_x_O_y_@TiO_2_-0.5 and Fe_x_O_y_@TiO_2_-0.35), and tested their performance in rhodamine 6G (R6G) photodegradation. X-ray diffraction (XRD) analysis showed that Fe_x_O_y_@TiO_2_-0.5 has the smallest crystallite size (16.8 nm), followed by Fe_x_O_y_@TiO_2_-0.5 (18.4 nm), Fe_x_O_y_/TiO_2_-0.35 (21.0 nm) and Fe_x_O_y_/TiO_2_-0.5 (19.0 nm), and X-ray photoelectron spectroscopy (XPS) suggested the decreasing percentage of Fe_3_O_4_ from 52.1% to 36.7%-47.2% after Ti-deposition treatment. The saturated magnetisms followed the order: Fe_x_O_y_@TiO_2_-0.5 > Fe_x_O_y_@TiO_2_-0.35 > Fe_x_O_y_/TiO_2_-0.5 > Fe_x_O_y_/TiO_2_-0.35. R6G photodegradation followed the first order kinetics and was slightly influenced by pH but significantly affected by initial photocatalyst concentration. Fe_x_O_y_/TiO_2_-0.35 achieved the highest removal efficiency for R6G (92.5%), followed by Fe_x_O_y_@TiO_2_-0.35 (88.97%), Fe_x_O_y_@TiO_2_-0.5 (60.49%) and Fe_x_O_y_/TiO_2_-0.5 (48.06%). Additionally, all these magnetic-TiO_2_-nanocomposites had satisfied magnetic recoverability and exhibited laudable reusability after 5-times reuse, even achieving higher R6G removal efficiencies from 97.30% to 98.47%. Our one-step method took only 75 min for nanocomposite synthesis, 90 min less than conventional two-step method, showing its feasibility as a practical method for magnetic-TiO_2_-nanocomposite synthesis in industrial application.

## 1. Introduction

Photocatalysis is one of most popular advanced oxidation processes (AOPs) in water treatment owning to its promising features of environment-friendly, generally non-selectivity and less secondary pollution [1]. In photocatalysis process, photocatalyst is the crucial component producing reactive oxygen species (ROS), *e*.*g*., OH radicals [2], during the reaction by stopping recombination of photo excited electron hole pair on the surface of the photocatalysts [3]. Among them, titanium dioxide (TiO_2_) is recognized as one of the best photocatalysts for its redox ability, high chemical and biological stability (particularly to photocorrosion), cost effectiveness and minor toxicity [3,4,5].

Bare TiO_2_ photocatalysts have some inherent shortages. As working within a narrow ultraviolet wavelength [6], TiO_2_ photocatalysts normally have a relatively low quantum efficiency and the most applied approach is minimizing the particulate size of TiO_2_ to achieve a large specific surface area [7]. Accordingly, most TiO_2_ catalysts are used in nano-scale [8,9], challenging the separation and recovery of these ultrafine particles after photocatalytic reaction. Considering their risks to the environment [10], recovering and reusing these TiO_2_ nano-photocatalysts is of great urgency. To solve this problem, some approaches are developed to support TiO_2_ nano-particulates with different anchors, *e*.*g*., glass and ceramic [11]. Nevertheless, the photoactivities of these fixed-bed systems suffer from the low ratio of mass transfer, comparing to the suspension systems [4]. Recent studies therefore propose the introduction of iron elements in TiO_2_ nano-photocatalysts as a promising method to achieve magnetic separation and harvesting [12]. Magnetic separation is more selective, efficient, and often much faster than centrifugation or filtration [13,14]. The key in magnetic separation is to generate strong magnetic forces on the target particles to overcome opposing forces, *e*.*g*. viscous drag, Brownian motion and sedimentation. Particulate size influences the magnetic forces [15], and decreasing the crystallite size from micrometers to nanometers significantly increases the magnetism by 100 to 1,000 times [16]. Thus, magnetic nanoparticulates (MNPs) have gained increasing attentions recently [17,18,19,20].

Coupling TiO_2_ photocatalysts with MNPs provides a group of novel nanophotocatalysts integrating the superior photodegradation performance of TiO_2_ and simple recovery by external magnetic fields. It has the potential to overcome the troubles in recovering and harvesting nanophotocatalysts after reuse. Generally, magnetic-TiO_2_-nanophotocatalysts are manufactured by functionalizing the surface of MNPs with one thin layer of TiO_2_ photocatalysts. Zhang *et al*. prepared magnetic-TiO_2_-nanophotocatalysts via wrapping TiO_2_ on montmorillonite and Fe_3_O_4_, achieving satisfied photodegradation rates and reusability for methylene blue [21]. Chen *et al*. synthesized three types of magnetic-TiO_2_-nanophotocatalysts, showing that Fe_2_O_3_@TiO_2_ had the highest adsorption efficiency (99%, 10 min) and Fe/TiO_2_ achieved the highest photodegradation efficiency (94%, 150 min) for methylene blue [22]. Magnetic-TiO_2_-nanophotocatalysts with different silver composing ratios could degrade 83.9% of methylene blue within 6 hours and the degradation efficiency remained 79% after 5-cycle reuse [23]. Although these magnetic-TiO_2_-nanophotocatalysts achieved satisfied photodegradation efficiencies and magnetic recoveries, they all require a complicated multiple-step synthesis and time-consuming pre-synthesis of MNPs or TiO_2_ precursor [5]. Alternatively, iron-composing is also used in TiO_2_ nanophotocatalysts to modify the bandgap energy of TiO_2_ and obtain a better photoactivity or extend the absorption spectrum [24,25]. The composing ratio of iron is normally less than 1% (mass ratio) owing to the declined photoactivity by excessive composing, and their magnetic recovery performance is not satisfied [26,27,28,29].

To the best of our knowledge, no previous research has reported simple or one-step synthesis processes to obtain iron-composed magnetic TiO_2_ nanophotocatalysts with high Fe atomic percentage (>20%) via coprecipitation method. In this study, we developed a facile approach to synthesize magnetic-TiO_2_-nanocomposites via one-step coprecipitation from iron and titanium salts. The synthesized magnetic-TiO_2_-nanocomposites achieved comparable photocatalytic degradation performance of rhodamine 6G (R6G) and had good durability after recovery and reuse. The main advantages of this method are: 1) simplified synthesis procedure without the need of pre-synthesizing MNPs or TiO_2_ precursor; 2) 55% less synthesis time comparing to two-step method; 3) cost-saving for fewer reagents in synthesis process.

## 2. Materials methods

### 2.1 Magnetic TiO_2_ synthesis

All the chemicals used in this study were of analytical grade and purchased from Sigma Aldrich (UK). One-step synthesis of TiO_2_-composing MNPs followed the previously reported coprecipitation method with modifications [30,31]. Briefly, 2 mL FeCl_3_ (1.0 M) and 1 mL FeCl_2_ (2.0 M) were gently mixed and added with 20 mL titanium isopropoxide (TTIP, 97%) and isopropanol (IPA, ≥99.7%). The mixture was kept shaking, to avoid agglomeration, with drop-by-drop addition of 45 mL NaOH (1.0 M) to control the crystallization rate, and continuously homologized for 30 min by ultrasound (60 kHz, LNGF175, Langford Electronics Ltd., UK). The synthesized brown-dark nanoparticles were harvested by a permanent magnet and rinsed with ultrapure water until the supernatant reached neutral pH (7.0). To investigate the impacts of Fe-composing ratio, two TTIP-IPA mixtures (2.4:17.6 and 3.4:16.6, m:m) were used to synthesize magnetic-TiO_2_-nanocomposites, named as Fe_x_O_y_/TiO_2_-0.5 (Fe:Ti = 0.5, mole:mole) and Fe_x_O_y_/TiO_2_-0.35 (Fe:Ti = 0.35, mole:mole). Details see S1 Table.

In comparison, two TiO_2_-coating magnetic nanophotocatalysts (core-shell), Fe_x_O_y_@TiO_2_-0.5 (Fe:Ti = 0.5, mole:mole) and Fe_x_O_y_@TiO_2_-0.35 (Fe:Ti = 0.35, mole:mole), were prepared using the conventional two-step synthesis method. Firstly, MNPs synthesis followed the reported protocol [32], except for Fe^2+^/Fe^3+^ ratio (1:1, mole:mole). The synthetized MNPs were washed with ultrapure water several times until pH was 7.0, and resuspended in 25 mL ultrapure water. Subsequently, 10 mL of MNPs suspensions were rinsed with 25 mL IPA three times for dehydration. Then, 25 mL IPA and 0.2 mL HCl (2.0 M) were added, gently mixed at 60 rpm, followed by rapidly adding TTIP of different volume (0.95 mL for Fe_x_O_y_@TiO_2_-0.5 and 1.35 mL for Fe_x_O_y_@TiO_2_-0.35) and keep shaking with drop-by-drop addition of 45 mL1.0 M NaOH (at least 45 minutes). After ultrasonic treatment for 30 min, another 1 mL NaOH (1.0 M) was added with an extra one-hour ultrasonic treatment.

All the synthesized magnetic-TiO_2_-nanocomposites were washed by ultrapure water until neutral pH. They were finally separated by magnet bar and dried overnight in a 60°C vacuum oven. After ground into powder, the granules were crystallized in a muffle furnace at 500°C for 4 hours.

### 2.2 Adsorption and photocatalysis experiment

Adsorption experiments were carried out in the dark to investigate R6G adsorption on magnetic-TiO_2_-nanocomposites. R6G was dissolved in ultrapure water to make series concentrations from 1 to 25 mg/L. The synthesized magnetic-TiO_2_-nanocomposites were added and the concentration was fixed as 0.4 g/L. The reaction system was kept continuous shaking (150 rpm) for 360 min and the water samples were collected at 1, 2, 3, 4, 5 and 6 hours. At each time point, the magnetic-TiO_2_-nanocomposites were removed by external magnetic field and the supernatant was collected for R6G analysis. To test the effects of pH on R6G adsorption, R6G solution was adjusted to pH≈3, 7 and 10 by adding HCl (1.0 M) or NaOH (1.0 M), respectively.

R6G photodegradation was carried out in 6-well plates (Costar, UK), with a 20-watt UV lamp (253 nm) as the irradiation source. Initial R6G concentration was 10 mg/L, and the concentration of magnetic-TiO_2_-nanocomposites was 0.4 g/L. During the 6-hour degradation experiment, the mixture was kept shaking at 150 rpm, and 250 μL of samples were collected at, 1, 2, 3, 4, 5 and 6 hours. After centrifugation at 10,000 rpm for 1 min, the supernatant was collected for R6G analysis. To test the effect of light wavelength on the photocatalytic degradation of R6G, 460-nm and 540-nm light was also used, following the same procedure described above. For the effects of pH and nanocomposite concentration on photocatalytic efficiency, the pH of the reaction system was adjusted to 3.0, 7.0 and 10.0 by adding HCl (1.0 M) or NaOH (1.0 M), and the concentrations of magnetic-TiO_2_-nanocomposites were set as 0.2, 0.4 and 0.8 g/L. For each experiment, a blank control without UV light was set up and all the adsorption and photodegradation experiments were carried out in triplicates.

To test the durability of the synthesized magnetic-TiO_2_-nanocomposites, they were recovered by a magnetic bar after 4-hour photodegradation of R6G, washed by ultrapure water for three times, dried at room temperature for 10 min, and finally added into fresh R6G for reuse, following the same procedure as described above. All the magnetic-TiO_2_-nanocomposites were recovered and reused at least five times.

### 2.3 Chemical analysis

Fe and Ti oxidation phases of the synthesized magnetic-TiO_2_-nanocomposites were analysed by X-ray photoelectron spectroscopy (XPS) via an ESCALAB 250Xi (Thermo Fisher, UK), using AlKα X-ray radiation (*hv* = 1486.6 eV, 1500 W) for excitation. X-ray diffraction (XRD) analysis was performed on an X' Pert PRO MPD diffractometer (PANalytical, Netherlands) equipped with Cu-Kα radiation at 40 kV and 40 mA. Surface morphologies were analysed using transmission electron microscopy (TEM, Tecnai G2 F20, USA). A vibrating sample magnetometer (VSM, Lake Shore 7410, USA) was used to measure the magnetism of the synthesized magnetic-TiO_2_-nanocomposites. Energy dispersive spectroscopy (EDS) analysis was conducted by a field emission scanning electron microscope (Quanta 450F, FEI, USA) with a voltage of 30 V. The Brunauer-Emmett-Teller (BET) surface area was measured by N_2_ physisorption on an Autosorb iQ-C (Quantachrome Instruments, USA). The concentrations of MNPs and magnetic-TiO_2_-nanocomposites were determined by gravimetric method. R6G concentration was determined by at 540 nm (excitation at 350 nm) by a multimode plate reader (FLUOstar Omega, Germany).

### 2.4 Data analysis

All the data were presented in terms of mean ± standard deviation. The removal efficiency (%) of R6G was calculated by the equation (*C*_*0*_-*C*_*t*_)/*C*_*0*_, where *C*_*0*_ is the initial concentration of R6G in the solution (mg/L) and *C*_*t*_ is the R6G concentration at time *t* (mg/L). Data of XPS and XRD were analysed by XPSpeak41 and Jade 6.5, respectively. The analysis of variance (ANOVA) was carried out via SPSS 20.0 for windows, and the significant level is *p*<0.05.

R6G degradation or removal efficiency was calculated as the ratio of residual R6G to initial R6G in each treatment. The R6G degradation kinetics was calculated according to the first-order reaction, ln(*C*_*t*_/*C*_*0*_) = -*k*_*obs*_*t*. Here, *k*_*obs*_ is the photodegradation rate constant (h^-1^). Both Langmuir and Freundlich isotherm models were used to assess the adsorption capacity of the synthesized magnetic-TiO_2_-nanocomposites, as expressed in the following equations:

$$Q=Q_{max}\frac{K_{L}C}{1+K_{L}C}$$ (1)

$$Q=K_{F}C^{1/n}$$ (2)

Here, *Q* is the amount of R6G adsorbed on magnetic-TiO_2_-nanocomposites (mg/g), and *C* is the equilibrium concentration of R6G in aqueous phase (mg/L). For Langmuir isotherm, *Q*_*max*_ is the theoretical maximum adsorption capacity (mg/g) and *K*_*L*_ is the adsorption equilibrium constant. *K*_*F*_ and *n* are the constants of Freundlich model.

To distinguish the different pathway of R6G loss by different magnetic-TiO_2_-nanocomposites *via* adsorption, photolysis and photocatalysis, the changes of R6G concentrations in different treatments were calculated by Eqs (3) to (8).

$${Total}_{0.2}={PL}_{0.2}+{AD}_{0.2}+{PC}_{0.2}$$ (3)

$${Total}_{0.4}={PL}_{0.4}+{AD}_{0.4}+{PC}_{0.4}$$ (4)

$${Total}_{0.8}={PL}_{0.8}+{AD}_{0.8}+{PC}_{0.8}$$ (5)

$$PAD=(\frac{{AD}_{0.2}}{{Total}_{0.2}}+\frac{{AD}_{0.4}}{{Total}_{0.4}}+\frac{{AD}_{0.8}}{{Total}_{0.8}})/3$$ (6)

$$PRL=(\frac{{PL}_{0.2}}{{Total}_{0.2}}+\frac{{PL}_{0.4}}{{Total}_{0.4}}+\frac{{PL}_{0.8}}{{Total}_{0.8}})/3$$ (7)

$$PRC=(\frac{{PC}_{0.2}}{{Total}_{0.2}}+\frac{{PC}_{0.4}}{{Total}_{0.4}}+\frac{{PC}_{0.8}}{{Total}_{0.8}})/3$$ (8)

Here, “0.2”, “0.4” and “0.8” indicates different treatments with initial magnetic-TiO_2_-nanocomposites concentration of 0.2, 0.4 and 0.8 mg/L, respectively. AD is the R6G loss in treatments with magnetic-TiO_2_-nanocomposites but no UV-irradiation. PL is the R6G loss in treatments with UV-irradiation but no magnetic-TiO_2_-nanocomposites. Total is the R6G loss in treatments with both magnetic-TiO_2_-nanocomposites and UV-irradiation. PC refers to the contribution of photocatalysis to R6G loss as calculated in Eqs (3)–(5). *PAD, PRL* and *PRC* refers to the average percentage of adsorption, photolysis and photocatalysis in R6G loss, respectively.

## 3. Results and discussion

### 3.1 Characteristics of magnetic-TiO_2_-nanocomposites

After crystallization, all the four types of magnetic-TiO_2_-nanocomposites had a red-brown colour (S1 Fig). TEM images (S2 Fig) illustrated that all the magnetic-TiO_2_-nanocomposites had a typical solid particle structure, irregular ball or rod. Their crystallite size ranged from 10–100 nm, fitting well with the critical size suggested for effective magnetic separation (~50 nm) [16]. The morphologies of these particles were similar to that of Fe(III)-composed TiO_2_ particles synthesized by previous work [33]. It is worth mentioning that the one-step synthesis of Fe_x_O_y_/TiO_2_ nanocomposites took only 75 min, 55% less than that of Fe_x_O_y_@TiO_2_ nanocomposites synthesized by conventional two-step method (165 min). EDS results (S3 Fig and S2 Table) showed different Fe/Ti ratios of synthesized magnetic-TiO_2_-nanocomposites, which were 0.55 (Fe_x_O_y_/TiO_2_-0.5), 0.34 (Fe_x_O_y_/TiO_2_-0.35), 0.40 (Fe_x_O_y_@TiO_2_-0.5) and 0.36 (Fe_x_O_y_@TiO_2_-0.35), respectively, consistent with the theoretical data. The BET surface area of Fe_x_O_y_/TiO_2_-0.5, Fe_x_O_y_/TiO_2_-0.35, Fe_x_O_y_@TiO_2_-0.5 and Fe_x_O_y_@TiO_2_-0.35 was 2.9, 7.2, 42.1 and 31.3 m^2^/g, showing significant higher surface areas of Fe_x_O_y_@TiO_2_ nanocomposites (two-step synthesis) than Fe_x_O_y_/TiO_2_ nanocomposites (one-step synthesis).

**Fig 1:**
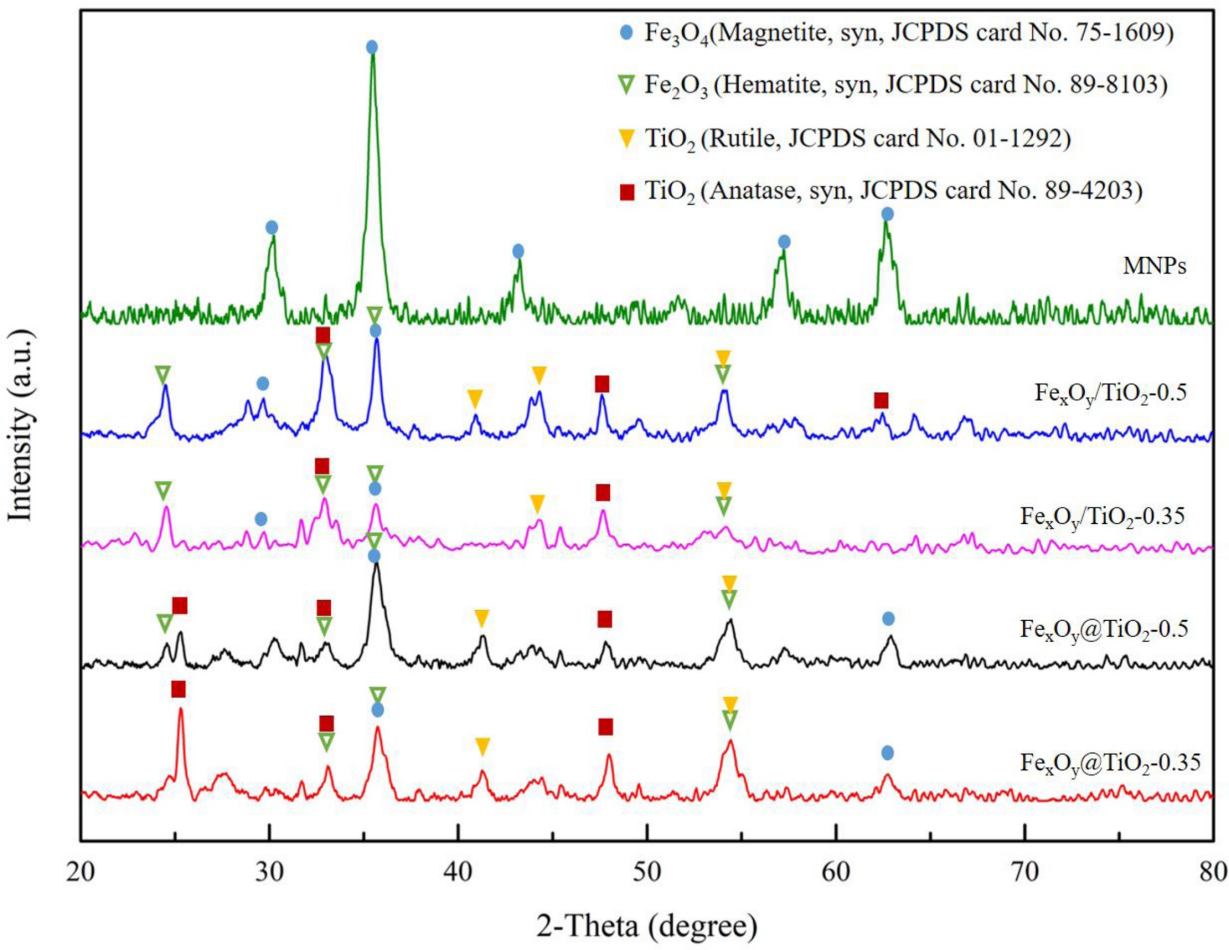
XRD patterns of MNPs and magnetic-TiO_2_-nanocomposites. Characteristic peaks of magnetite, hematite, rutile TiO_2_ and anatase TiO_2_ are marked with blue circle, green triangle, yellow triangle and red square, respectively.

Fig 1 shows the XRD patterns of MNPs and magnetic-TiO_2_-nanocomposites. MNPs had sharp and intense peaks of magnetite (Joint Committee of Powder Diffraction Standard, JCPDS, card No. 75–1609), indicating its composition of an iron oxide Fe_3_O_4_. From Scherrer’s formula [34], the average crystallite size of MNPs was 13.2 nm, calculated from the most intense peak corresponding to (311). For magnetic-TiO_2_-nanocomposites, the peaks of TiO_2_ were also observed at 2*θ* = 25.30° (101), 33.45° (110), 48.04° (200) and 62.68° (204) (Anatase, syn, card No. 89–4203), and at 2*θ* = 27.51° (110), 36.04° (101), 41.19° (111), 44.14° (210) and 54.23° (211) (Rutile, card No. 01–1292). It suggested the successful coupling of TiO_2_ and MNPs, although there was some slight phase transformation presence in MNPs. The crystallite size of these magnetic TiO_2_ was 19.0 nm (Fe_x_O_y_/TiO_2_-0.5), 21.0 nm (Fe_x_O_y_/TiO_2_-0.35), 18.4 nm (Fe_x_O_y_@TiO_2_-0.5) and 16.8 nm (Fe_x_O_y_@TiO_2_-0.35), all of which were larger than MNPs. It is worth noting that all the magnetic-TiO_2_-nanocomposites had the signals of hematite (syn, card No. 89–8103) at the peaks 2*θ* = 24.22° (012) 33.24° (104), 35.74° (110), 54.22° (116) and 62.63° (214), suggesting the altered valence state of iron by titanium.

**Fig 2:**
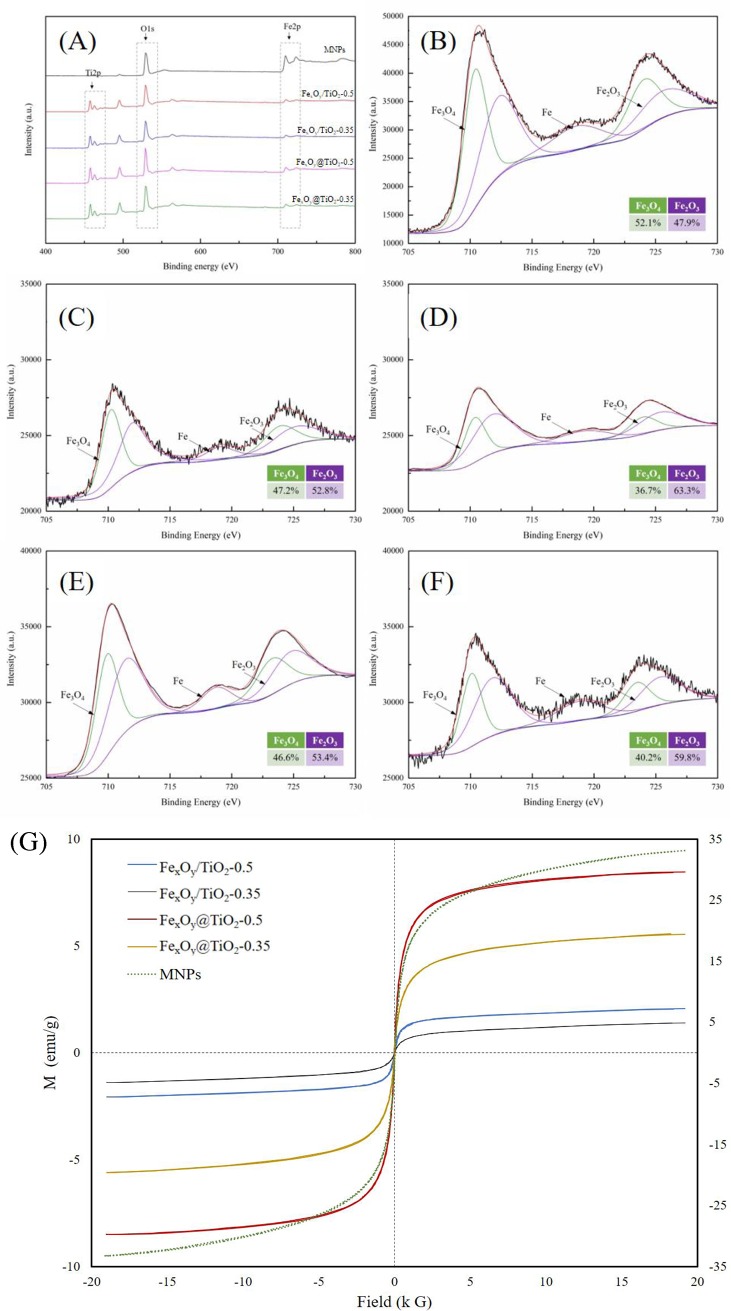
Characteristics of magnetic-TiO_2_-nanocomposites. (A) XPS spectra of the synthesized MNPs and magnetic-TiO_2_-nanocomposites. Fe2p region spectra: MNPs (B), Fe_x_O_y_/TiO_2_-0.5 (C), Fe_x_O_y_/TiO_2_-0.35 (D), Fe_x_O_y_@TiO_2_-0.5 (E) and Fe_x_O_y_@TiO_2_-0.35 (F). (G) Magnetization curve for magnetic-TiO_2_-nanocomposites.

The elemental composition of the synthesized MNPs and magnetic-TiO_2_-nanocomposites was analysed by XPS (Fig 2A). All the materials had iron and oxygen as core elements, owing to the main peaks of Fe 2p (710.3 to 710.7 eV) and O 1s (529.3 to 529.9 eV). One additional Ti 2p peak (457.8 to 457.9 eV) appeared in the XPS spectra of magnetic-TiO_2_-nanocomposites. Fig 2B to 2F further show the Fe 2p bands in the binding energy region of 705–730 eV. MNPs showed the strongest intensity, followed by Fe_x_O_y_@TiO_2_ nanocomposites. All the spectra of magnetic-TiO_2_-nanocomposites could fit into two Fe 2p3/2 peaks (around 710 eV and 723 eV) and two Fe 2p1/2 peaks (around 711 eV and 725 eV) [35,36], suggesting the presence of both Fe_3_O_4_ and Fe_2_O_3_ [37]. Furthermore, the satellite peak at 719 eV is the main identity of Fe_2_O_3_ or FeO [38]. Accordingly, Fe_3_O_4_ accounted for 52.1% of the total iron oxides in MNPs, decreasing to 36.7%-47.2% in magnetic-TiO_2_-nanocomposites. In addition, nanocomposites with higher Ti-deposition ratio had relatively higher proportion of Fe_2_O_3_, further proving the change of iron valence state by titanium.

The saturation magnetism (*M*_*s*_) of Fe_x_O_y_/TiO_2_-0.5, Fe_x_O_y_/TiO_2_-0.35, Fe_x_O_y_@TiO_2_-0.5 and Fe_x_O_y_@TiO_2_-0.35 was 2.06, 1.40, 8.45 and 5.56 emu/g, respectively, as illustrated in Fig 2G. The coercivity (*H*_*c*_) and remanence magnetization (*M*_*r*_) of all the magnetic-TiO_2_-nanocomposites were zero, indicating that the hysteresis loops were coincident completely and these nanocomposites dispersed well in the absence of external magnetic field. Generally, the saturation magnetisms of Fe_x_O_y_@TiO_2_ nanocomposites were higher than Fe_x_O_y_/TiO_2_ ones, explained by the incomplete coordination of iron atoms in the Fe_x_O_y_/TiO_2_ nanocomposites [5]. Additionally, the saturation magnetism increased with the Fe/Ti ratio, owing to the strong link between *M*_*s*_ and the percentage of Fe_3_O_4_ [39]. The saturation magnetisms of our synthesized magnetic-TiO_2_-nanocomposites fell in the range of previously studied magnetic nanophotocatalysts (0.54–42.2 emu/g, Table 1), indicating their comparable magnetic recovery or separation efficiency.

**Table 1:** Saturation magnetisms of reported magnetic nanophotocatalysts.

Nanophotocatalyst	*M*_*s*_ (emu/g)	Reference
Ce/TiO_2_/NiFe_2_O_4_/diatomite	6.8	[40]
NiFe_2_O_4_/diatomite	8.0	[40]
Fe_3_O_4_/ZnO/Ag_3_VO_4_/AgI	6.26	[41]
Fe_3_O_4_/TiO_2_	7.5–42.2	[42]
Fe_3_O_4_@SiO_2_/TiO_2_	10.0–20.2	[42]
Fe_3_O_4_@SiO_2_@Iodine/TiO_2_	0.54–11.56	[43]
Fe_3_O_4_@SiO_2_@TiO_2_	15.9	[44]
Fe_3_O_4_@SiO_2_@TiO_2_/Pd	7.5	[44]
Fe_2_O_3_-TiO_2_	0.55–2.14	[45]
Fe_x_O_y_/TiO_2_	1.40–2.06	This work
Fe_3x_O_y_@TiO_2_	5.56–8.45	This work

### 3.2 R6G adsorption isotherm

The adsorption of organic molecules on catalysts is an important process during photocatalysis [46], which is related to the R6G photocatalytic degradation efficiency by magnetic-TiO_2_-nanocomposites. Our results indicated that all the magnetic-TiO_2_-nanocomposites synthesized exhibited a significant R6G adsorption (S4 Fig). R6G aqueous concentrations decreased dramatically in the first 50 min and became stable in 1–2 hours. After 6-hour adsorption, Fe_x_O_y_@TiO_2_-0.5 had the highest R6G adsorption capacity (1.65–18.62 mg/mg), followed by Fe_x_O_y_@TiO_2_-0.35 (1.72–16.6 mg/mg), Fe_x_O_y_/TiO_2_-0.5 (1.67–12.49 mg/mg) and Fe_x_O_y_/TiO_2_-0.35 (1.75–8.61 mg/mg). Bare MNPs showed similar adsorption performance comparing to the synthesized magnetic-TiO_2_-nanocomposites with no significant difference (data not shown, p > 0.05), indicating that the presence of Fe_3_O_4_ and Fe_2_O_3_ contributed to R6G adsorption but did not change the adsorption performance.

**Fig 3:**
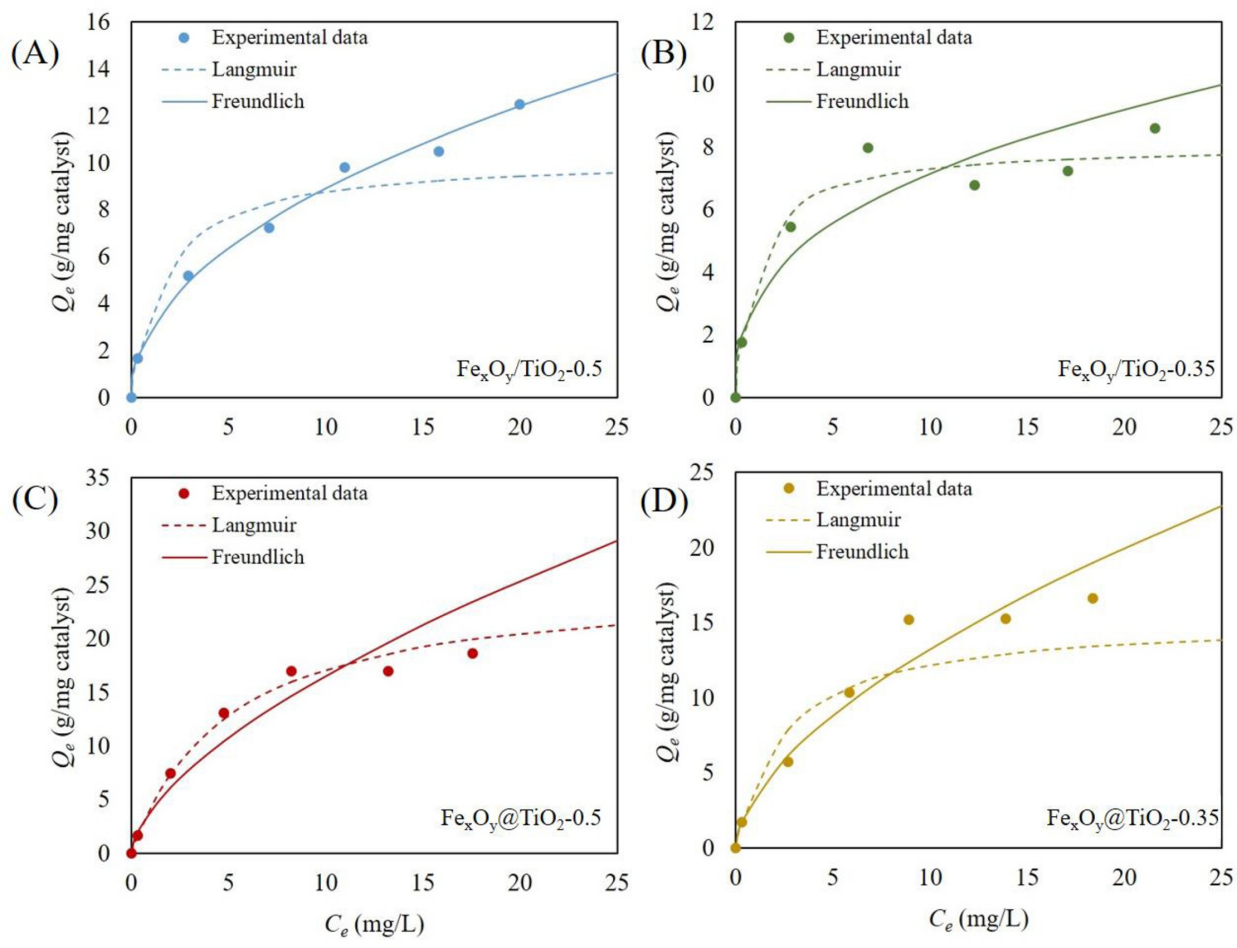
Langmuir (dotted line) and Freundlich (solid line) adsorption isotherms of R6G on the synthesized magnetic-TiO_2_-nanocomposites. (A) Fe_x_O_y_/TiO_2_-0.5, (B) Fe_x_O_y_/TiO_2_-0.35, (C) Fe_x_O_y_@TiO_2_-0.5, (D) Fe_x_O_y_@TiO_2_-0.35. Dots represent experimental data. Experimental conditions: magnetic-TiO_2_-nanocomposites concentration, 0.4 g/L; initial R6G concentration from 1 to 25 g/L; pH, 7.0.

Fig 3 illustrates the satisfied fitness of the four magnetic-TiO_2_-nanocomposites with Langmuir and Freundlich isotherms, respectively. From the isotherm parameters (Table 2), Langmuir adsorption described the adsorption mechanisms better except for Fe_x_O_y_/TiO_2_-0.5 according to *R*^2^ of each nanocomposite. Accordingly, Fe_x_O_y_@TiO_2_ nanocomposites presented a better adsorption performance than Fe/Ti ones. Previous studies [47,48,49] suggested that the molecular adsorption on catalysts is involved in the surface complexation and ion exchange between the catalyst surface and the adsorbates. The functional groups located on the surface of TiO_2_ include TiOH_2_^+^, TiOH and TiO^−^[50], which are more abundant than those on the surface of iron oxides [51]. The adsorption of R6G on nanocomposites is therefore dependent on the proportion of TiO_2_ on the surface, which is higher in Fe_x_O_y_@TiO_2_ nanocomposites.

**Table 2:** Parameters of *Q*_*max*_ and *K*_*L*_ in Langmuir isotherm and *K*_*F*_ and 1/*n* in Freundlich isotherm.

Isotherm	Langmuir			Freundlich		
Parameters	*Q*_*max*_ (mg/g)	*K*_*L*_	*R*^2^	*K*_*F*_ (mg/g)	1/*n*	*R*^2^
**Fe_x_O_y_/TiO_2_-0.5**	10.21	0.5883	0.9863	2.92	0.4831	0.9958
**Fe_x_O_y_/TiO_2_-0.35**	8.09	0.9237	0.9943	3.10	0.3532	0.9036
**Fe_x_O_y_@TiO_2_-0.5**	25.51	0.2030	0.9997	3.96	0.6204	0.9459
**Fe_x_O_y_@TiO_2_-0.35**	15.29	0.3893	0.9862	3.41	0.5900	0.9816

In the present study, pH change from 3 to 10 did not significantly affect the R6G adsorption on magnetic-TiO_2_-nanocomposites (S5 Fig, *p*>0.05). pH has been considered as one of the most important operational parameters in many photocatalytic systems, significantly affecting the photocatalytic efficiency *via* changing the structure and adsorption-desorption process of adsorbates. As the point of zero charge (PZC) for TiO_2_ is 6.25 [5], the changes in surface charge of TiO_2_ at various pH values are expressed in Eqs (9) and (10) [50]. At pH lower than PZC, the increasing proportion of TiOH_2_^+^ led to the positively charged TiO_2_ surface, whereas the negatively charged TiO^-^ occurs at pH>PZC. Meanwhile, R6G has a logarithmic acid dissociation constant (*pKa*) of 6.13, similar as the PZC for TiO_2_. At pH>6.13, R6G is primarily in molecular state as the free electron pair on the primary amine group transfer to the aromatic rings and behave hydrophobic, but change to hydrophilic at lower pH when the protonation of nitrogen carries on in the secondary amine group of the xanthene ring [52]. Accordingly, the electrostatic force interactions between TiO_2_ surface and R6G were reported to be weak repulsion or attraction in a broad range of pH [53], showing similar adsorption performance.

$$TiOH+H^{+}\to Ti{OH}_{2}^{+}\;(pH<6.25)$$ (9)

$$TiOH+{OH}^{-}\to TiO^{-}+H_{2}O\;(pH>6.25)$$ (10)

### 3.3 Contribution of adsorption, photolysis and photocatalysis to R6G degradation

**Fig 4:**
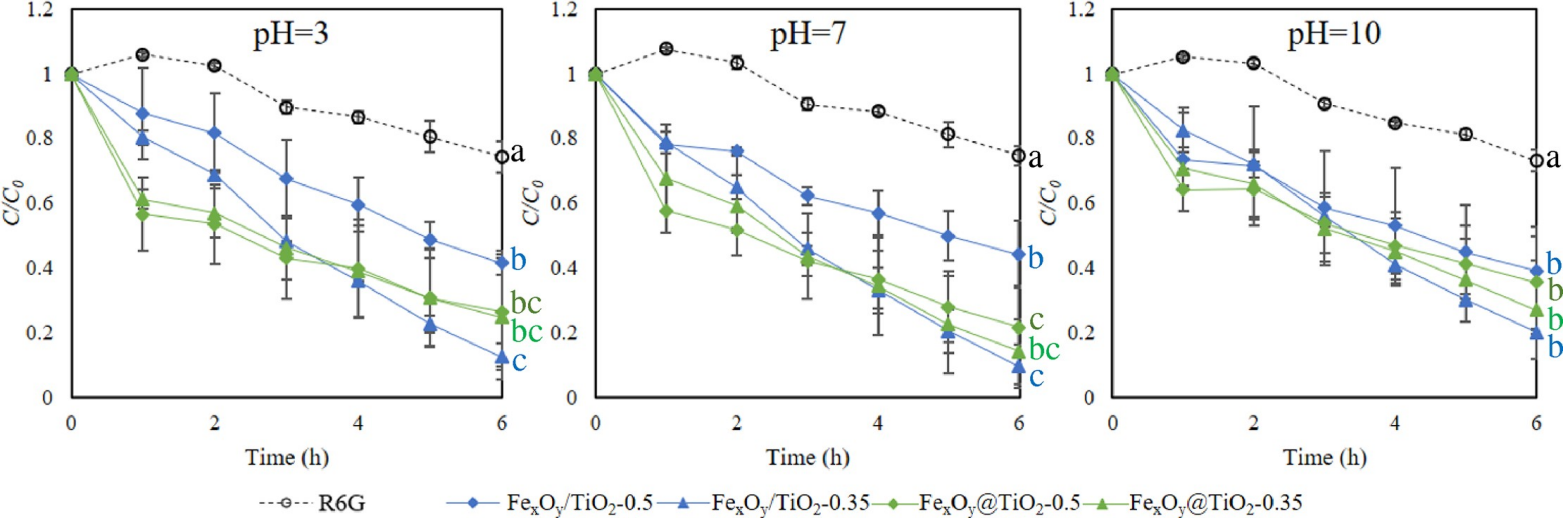
Impacts of pH on R6G photocatalysis performance. (A) pH = 3, (B) pH = 7, (C) pH = 10. Dotted lines represent the change of R6G concentration under UV-irradiation without magnetic-TiO_2_-nanocomposites, and solid lines represent R6G photodegradation dynamics under UV-irradiation with magnetic-TiO_2_-nanocomposites. Experimental conditions: UV-irradiation, 253 nm (20 W); magnetic-TiO_2_-nanocomposites concentration, 0.4 g/L; initial R6G concentration, 10 mg/L. Different small letters after each line indicate significant difference (Duncan’s test, p < 0.05) among treatments (n = 3).

Under all pH conditions, R6G exhibited limited removal without light (approximately 14.1%, S6 Fig) which was significantly lower than those with 253-nm UV-irradiation (averagely 73.58%, p<0.01, Fig 4). In addition, the R6G degradation efficiency with 460-nm or 540-nm irradiation (S7 Fig) was similar as that without light. It proved the significant contribution of photocatalysis to R6G degradation comparing to adsorption, and UV irradiation wavelength is a critical factor triggering R6G photodegradation with the aid of magnetic-TiO_2_-nanocomposites. It is worth mentioning that there was neglectable R6G degradation in treatments with bare MNPs (data not shown), proving that neither Fe_3_O_4_ nor Fe_2_O_3_ contributed to R6G photocatalysis.

However, the loss of R6G might be explained by either adsorption, photolysis or photocatalysis as previously reported [46], which needs to be distinguished to evaluate the actual R6G photocatalysis performance.

S8 Fig summarizes the percentage of R6G loss during degradation, attributing to adsorption, photolysis and photocatalysis, respectively. Their contribution obviously varied across magnetic-TiO_2_-nanocomposite types and concentrations. For instance, photolysis accounted for averagely 45.5% of the total R6G loss by Fe_x_O_y_/TiO_2_-0.5, followed by adsorption (averagely 34.0%) and photocatalysis (averagely 20.5%). In contrast, photocatalysis had a higher contribution to R6G loss by Fe_x_O_y_@TiO_2_-0.5 (averagely 31.3%). With the increasing TiO_2_ proportion, the contribution of photocatalysis significantly increased to 58.3% for Fe_x_O_y_/TiO_2_-0.35 and 63.1% for Fe_x_O_y_@TiO_2_-0.35, which were close to Chen’s report (56.7%-67.1%) [23]. These results indicated that the photocatalytic performance of the synthesised magnetic-TiO_2_-nanocomposites increased with the Ti/Fe ratio. Since the photocatalytic active sites are mainly located on TiO_2_ instead of iron oxides [43], higher percentage of TiO_2_ in magnetic-TiO_2_-nanocomposites increases the photocatalysis capability. Iron- composing benefits the photocatalytic performance of TiO_2_ because transition metal ions can act as shallow charge traps in the crystal structure, inhibiting the recombination of electron-hole pairs and prolonging their lifetime [54]. Although the impacts of transition metals on TiO_2_ catalytic performance strongly depend on the composing ratio and are reported to peak at low level, all the previous studies focused on the low Fe-composing ratio, normally less than 10 at.% [33,55]. Our synthesized magnetic-TiO_2_-nanocomposites had the high Fe:Ti ratio (Fe-atomic percentage ranging from 25.4–35.5%). For the first time, our results suggested magnetic-TiO_2_-nanocomposites with high Fe-composing ratio still have satisfied photocatalysis capability.

### 3.4 Photocatalytic performance of magnetic-TiO_2_-nanocomposites

R6G degradation curves in different treatments with the synthesized magnetic-TiO_2_-nanocomposites are illustrated in Fig 4. In blank controls (no magnetic-TiO_2_-nanocomposites), R6G concentration decreased only 25.6%, 25.2% and 26.9% under acidic (pH≈3), neutral (pH≈7) and basic (pH≈10) conditions after 6-hour experiment. In contrast, treatments with magnetic-TiO_2_-nanocomposites achieved much higher removal efficiency, averagely 73.6% (pH≈3), 77.6% (pH≈7) and 69.5% (pH≈10), respectively. Especially, Fe/TiO_2_-0.35 was the most efficient one (removal efficiency 85.9%), followed by Fe@TiO_2_-0.35 (78.0%), Fe@TiO_2_-0.5 (72.1%) and Fe/TiO_2_-0.5 (58.3%).

Our results showed slightly higher R6G photodegradation efficiencies under neutral pH conditions than acidic and basic treatments. It might be explained by the higher R6G adsorption on magnetic-TiO_2_-nanocomposites as mentioned above. As adsorption is a key step for photocatalysis [46], more R6G absorbed on magnetic-TiO_2_-nanocomposites under neutral pH therefore contribute to higher R6G removal. A similar result was also reported that the highest efficiency was obtained at the neutral condition (pH = 7.4) during the phenol photodegradation process with TiO_2_ as photocatalyst [50].

The impacts of magnetic-TiO_2_-nanophotocatalyst concentration on R6G photocatalysis were investigated under neutral pH condition (pH≈7), as shown in S9 Fig. For single kind of nanophotocatalyst, the initial concentration influence to R6G degradation significantly. For instance, the R6G removal efficiency in Fe_x_O_y_/TiO_2_-0.5 treatment was 31.86% (0.2 g/L), 48.06% (0.4 g/L) and 39.09% (0.8g/L), respectively (p<0.05). It ranged from 74.63% to 92.50% for Fe_x_O_y_/TiO_2_-0.35 (p<0.05 except for 0.4 g/L vs. 0.8 g/L, p = 0.084), 40.72% to 60.49% for Fe_x_O_y_@TiO_2_-0.5 (p<0.05 except for 0.2 g/L vs. 0.4 g/L, p = 0.988) and 72.22% to 88.97% for Fe_x_O_y_@TiO_2_-0.35 (p<0.05). There was no significant difference in R6G removal efficiency between Fe/TiO_2_ and Fe_x_O_y_@TiO_2_ nanophotocatalysts (p = 0.329), hinting similar photocatalytic efficiency of magnetic-TiO_2_-nanocomposites synthesized by one-step and two-step methods.

### 3.5 R6G photodegradation kinetics

**Fig 5:**
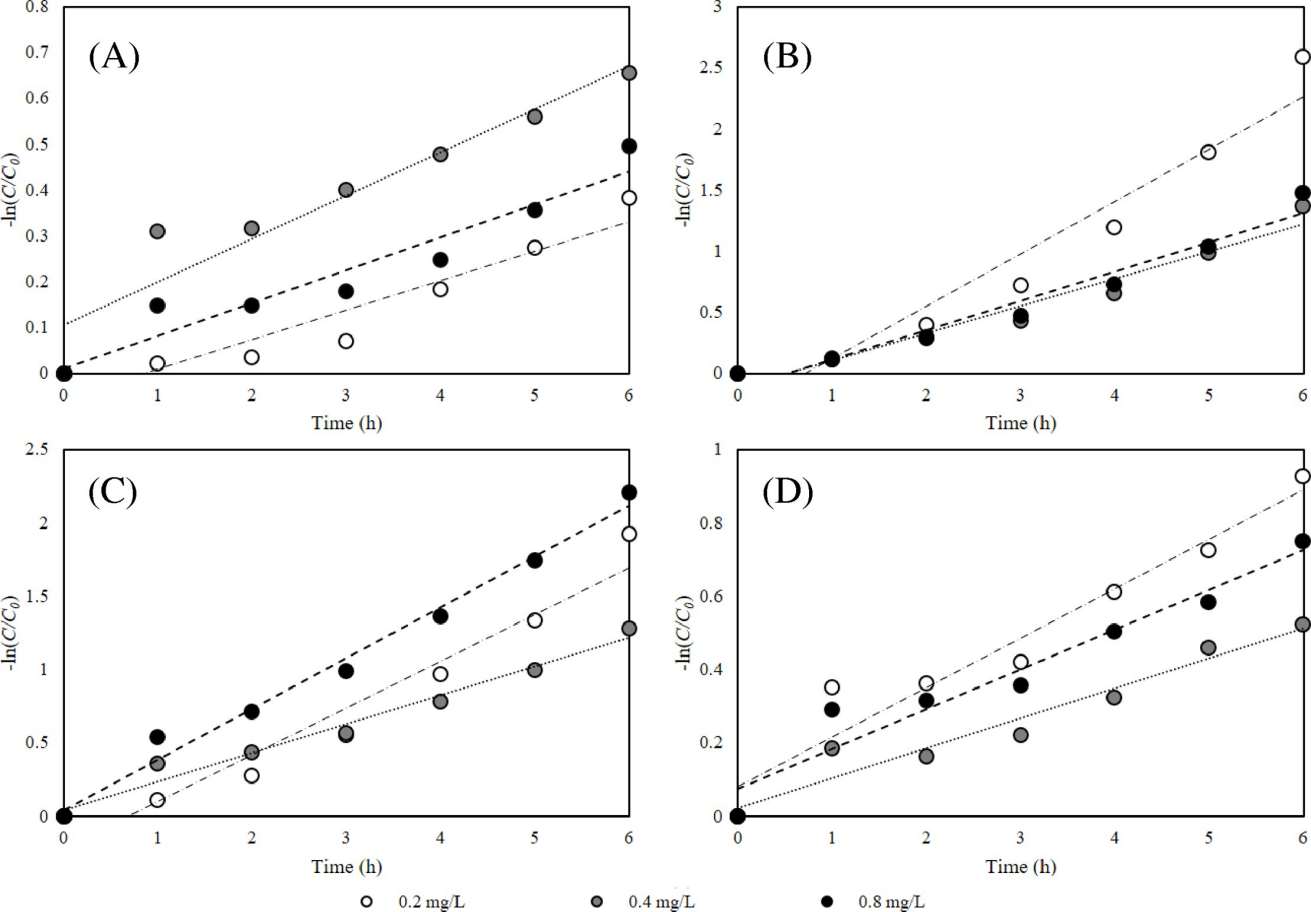
Impacts of initial magnetic-TiO_2_-nanocomposite concentration on R6G photodegradation kinetics. (A) Fe_x_O_y_/TiO_2_-0.5, (B) Fe_x_O_y_/TiO_2_-0.35, (C) Fe_x_O_y_@TiO_2_-0.5 and (D) Fe_x_O_y_@TiO_2_-0.35. Experimental conditions: UV-irradiation, 253 nm (20 W); initial R6G concentration, 10 mg/L; initial magnetic-TiO_2_-nanocomposites concentration, 0.2 mg/L, 0.4 mg/L and 0.8 mg/L, respectively; pH, 7.0.

The photocatalysis kinetics of R6G by different magnetic-TiO_2_-nanocomposites are illustrated in Fig 5. R6G degradation followed the first-order kinetics (correlation coefficient above 0.9), consistent with previous study [52]. For two-step synthesized magnetic-TiO_2_-nanocomposites, R6G photodegradation rate constant (*k*_*obs*_) ranged from 0.0816 to 0.1351 h^-1^ for Fe_x_O_y_@TiO_2_-0.5, increasing to 0.1954–0.3454 h^-1^ for nanophotocatalysts with higher Ti proportion (Fe_x_O_y_@TiO_2_-0.35). They were similar with the degradation rate constant of one-step synthesized magnetic-TiO_2_-nanophotocatalysts with no significant difference (p = 0.329), 0.0643–0.0939 h^-1^ for Fe_x_O_y_/TiO_2_-0.5 and 0.2225–0.4723 h^-1^ for Fe_x_O_y_/TiO_2_-0.35.

Among them, Fe_x_O_y_@TiO_2_-0.35 and Fe_x_O_y_/TiO_2_-0.35 nanocomposites showed higher photocatalytic activities than previously reported methylene blue degradation by magnetic-nanophotocatalyst Ni-Cu-Zn ferrite@SiO_2_@TiO_2_@Ag [23] (*k*_*obs*_ from 0.1326 to 0.2544 h^-1^). It is worth noting that the dosage of magnetic-TiO_2_-nanocomposites did not significantly change the *k*_*obs*_, possibly attributing to the decrease of surface active sites as nanophotocatalysts aggregate at elevated concentration, or the declined light utilization efficiency caused by the increasing opacity level and scattering of light at high nanocomposite concentration [56].

### 3.6 Reusability of magnetic-TiO_2_-nanophotocatalysts for R6G photodegradation

**Fig 6:**
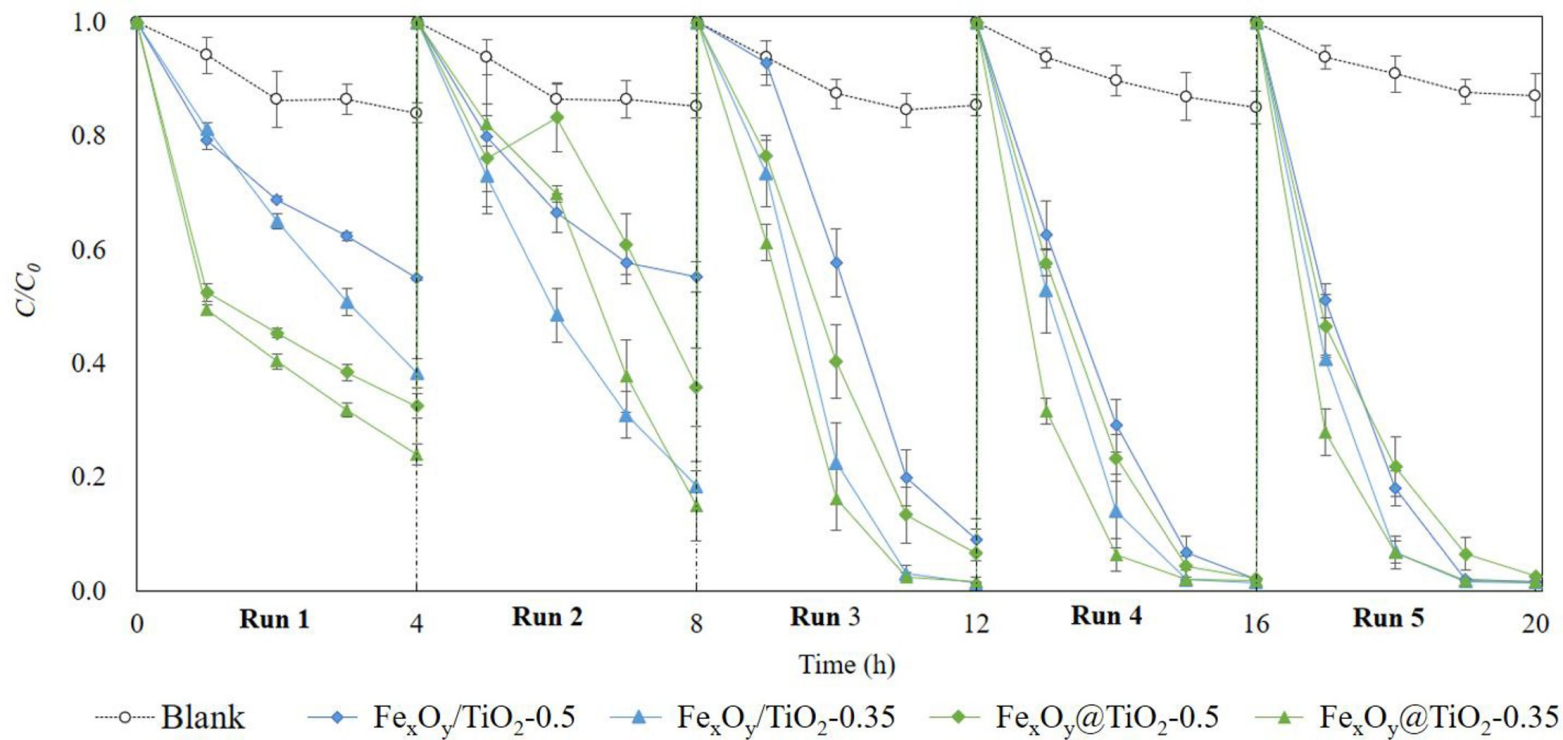
R6G degradation performance after reusing magnetic-TiO_2_-nanocomposites 5 times. Dotted lines represent R6G concentration dynamics in blank treatment without magnetic-TiO_2_-nanocomposites and solid lines represent R6G photodegradation dynamics under UV-irradiation with magnetic-TiO_2_-nanocomposites. Experimental conditions: UV-irradiation, 253 nm (20 W); initial magnetic TiO_2_ nanocomposites concentration, 0.4 g/L; initial R6G concentration, 10 mg/L; pH, 7.0.

Reusability is a critical factor for evaluating the practical feasibility of nanophotocatalysts in industrial applications. After recycling the synthesized magnetic-TiO_2_-nanocomposites and reusing them for R6G photocatalytic degradation five times, our results proved their satisfactory stability in photocatalysis, as illustrated in Fig 6. The photocatalytic activities of all the magnetic-TiO_2_-nanocomposites remained unchanged or even increased in all the cycles. After 5 cycles, the R6G removal efficiency was up to 97.30% to 98.47%, and there was no significant difference between the four magnetic-TiO_2_-nanocomposites. Additionally, R6G degradation still followed the first-order kinetics and the photodegradation rate constant *k*_*obs*_ was 0.1434–1.088 h^-1^ (Fe_x_O_y_/TiO_2_-0.5), 0.2380–1.1486 h^-1^ (Fe_x_O_y_/TiO_2_-0.35), 0.2554–0.9177 h^-1^ (Fe_x_O_y_@TiO_2_-0.5) and 0.3285–1.1411 h^-1^ (Fe_x_O_y_@TiO_2_-0.35), respectively. The increasing instead of decreasing *k*_*obs*_ during reuse was also reported by some previous studied and might be explained by the activation of photocatalytic active sites [57,58,59].

Numerous studies have attempted to synthesize and reuse different types of magnetic nanophotocatalysts in removing dyes, as listed in Table 3. For industrial application, both photocatalytic capability and recovery ratio are important to achieve cost-effectiveness. Nevertheless, none of the studies mentioned above have provided data support. Here, we evaluated the recovery ratio for all the synthesized magnetic-TiO_2_-nanocomposites, which were acceptable after 5-time reuse and achieved 68.83% for Fe_x_O_y_/TiO_2_-0.5, 65.38% for Fe_x_O_y_/TiO_2_-0.35, 80.32% for Fe_x_O_y_@TiO_2_-0.5 and 77.84% for Fe_x_O_y_@TiO_2_-0.35. Our result indicated long life-cycles of these synthesized magnetic-TiO_2_-nanocomposites, and the recovery ratio might be further improved by increasing *M*_*s*_ to enhance magnetic separation efficiency and prolong their life-time.

**Table 3:** Photocatalytic performance of magnetic photocatalysts after reuse.

Catalyst or reaction system	Pollutants	Irradiation	Removal efficiency		Reuse	Reaction Time	Reference
			Fresh	Final			
Ni-Cu-Zn Ferrite@SiO_2_@TiO_2_@Ag	Methylene blue	Simulated sunlight	87.8%	46.2%	5	6 h	[23]
TiO_2_-FeZ and H_2_O_2_	Diclofenac	Simulated sunlight	ac. 89%	ac. 77%	5	60 min	[62]
Ce/TiO_2_/NiFe_2_O_4_/diatomite	Oxytetracycline	Visible light	98.0%	96.1%	6	4 h	[40]
Fe_3_O_4_@SiO_2_@TiO_2_/Pd-Ag	Rhodamine B	UV irradiation	96.1%	84.0%	6	40 min	[44]
Fe_3_O_4_@SiO_2_@TiO_2_/Pd	Rhodamine B	UV irradiation	84.0%	77.0%	5	40 min	[44]
Fe_3_O_4_@C@CdS	Rhodamine B	Visible light	ac. 95%	ac. 86%	6	50 min	[63]
Fe_3_O_4_@TiO_2_	Rhodamine B	UV irradiation	ac. 80%	ac. 74%	6	-	[64]
TiO_2_/montmorillonite/Fe3O4	Methylene blue	UV irradiation	94.0%	90.0%	6	80 min	[21]
γ-Fe_2_O_3_@SiO_2_@TiO_2_-Ag	Methyl orange	UV irradiation	84.5%	82.0%	18	60 min	[65]
ZnFe_2_O_4_/SrFe_12_O_19_	Methylene blue	UV-vis. irradiation	96.6%	70.6%	5	60 min	[66]
Carbon-nanotubes/Fe_3_O_4_-ZnO	Methyl orange	UV irradiation	ac. 100%	ac. 90%	2	45 min	[67]
Fe_3_O_4_@C@Cu_2_O	Rhodamine B	Visible light	ac. 100%	ac. 95%	6	-	[68]
Barium-ferrite/SiO_2_/TiO_2_	Procion red	UV irradiation	ac. 60%	ac. 62%	3	80 min	[69]
Heat-treated-BF/SiO_2_/TiO_2_	Procion red	UV irradiation	ac. 80%	ac. 80%	3	80 min	[69]
Ag-TiO_2_/SiO_2_/Fe_3_O_4_	Orange II	UV irradiation	ac. 90%	ac. 80%	3	20 min	[70]
Fe_x_O_y_/TiO_2_-0.5	R6G	UV irradiation	45.0%	98.4%	5	6 h	This work
Fe_x_O_y_/TiO_2_-0.35	R6G	UV irradiation	61.6%	98.5%	5	6 h	This work
Fe_x_O_y_@TiO_2_-0.5	R6G	UV irradiation	67.4%	97.3%	5	6 h	This work
Fe_x_O_y_@TiO_2_-0.35	R6G	UV irradiation	75.9%	98.3%	5	6 h	This work

Of all the concerns to apply nanophotocatalysts for industrial applications, including the extension of excitation wavelength, viability of magnetic separation, photocatalytic activity and cost-effectiveness in nanocomposite synthesis, our study attempted to reduce the synthesis time and chemical consumption by one-step facile synthesis. More studies can be introduced from this basis to target other objectives. For instance, iron-composing was reported to extend the excitation wavelength of TiO_2_ nanophotocatalysts [60] or improve their photocatalytic efficiency [61], and the magnetism of iron-composing TiO_2_ nanophotocatalysts can be altered by synthesis modification. Accordingly, our one-step facile synthesis of magnetic-TiO_2_-nanophotocatalysts with high iron-composing ratio provides opportunities for synthesizing similar magnetic-TiO_2_-nanocomposites with new features for further industrial applications.

## 4. Conclusion

In the present study, magnetic-TiO_2_-nanocomposites with high iron-composing g ratio were prepared by a one-step coprecipitation method without any pre-synthesized precursor. Comparing to conventional two-step synthesized TiO_2_-coating magnetic-nanocomposites with MNPs as precursor, we successfully decreased the synthesis time from 165 minutes to 75 minutes. Our results also proved that one-step synthesised magnetic-TiO_2_-nanocomposites had satisfied R6G adsorption capability, photodegradation efficiency and photodegradation rate constant. The photocatalytic performance was dependent on Fe/Ti atomic ratio, and Fe_x_O_y_/TiO_2_-0.35 was the most feasible nanophotocatalysts for industrial application. With facile synthesis process, comparable photocatalytic capability, acceptable viability of magnetic separation and satisfactory reusability, this synthesis method provides an alternative approach to prepare magnetic-TiO_2_-nanophotocatalysts feasible for industrial practices.

## Supporting information

S1 Table Parameters for synthesizing magnetic-TiO_2_-nanocomposites. (DOCX)

S2 Table Fe/Ti ratio of synthesizing magnetic-TiO_2_-nanocomposites calculated by EDS results. (DOCX)

S1 Fig Photographs of synthesizing and harvesting the synthesized magnetic-TiO_2_-nanocomposites. (DOCX)

S2 Fig TEM images of (A) Fe_x_O_y_/TiO_2_-0.5, (B) Fe_x_O_y_/TiO_2_-0.35 (C) Fe_x_O_y_@TiO_2_-0.5 and (D) Fe_x_O_y_@TiO_2_-0.35. Scale bar: 100 nm.
(DOCX)

S3 Fig Energy dispersive spectroscopy (EDS) pattern of synthesized magnetic-TiO_2_-nanocomposites. (A) Fe_x_O_y_/TiO_2_-0.5, (B) Fe_x_O_y_/TiO_2_-0.35 (C) Fe_x_O_y_@TiO_2_-0.5 and (D) Fe_x_O_y_@TiO_2_-0.35.
(DOCX)

S4 Fig R6G adsorption kinetics on the synthesized magnetic-TiO_2_-nanocomposites. (A) 1 mg/L, (B) 5 mg/L, (C) 10 mg/L, (D) 15 mg/L, (E) 20 mg/L, (F) 25 mg/L. Experimental conditions: magnetic-TiO_2_-nanocomposites concentration, 0.4 g/L; pH, 7.0. Different small letters after each line indicate significant difference (Duncan’s test, p < 0.05) among treatments (n = 3).
(DOCX)

S5 Fig Impacts of pH on R6G adsorption on the synthesized magnetic-TiO_2_-nanocomposites. Experimental conditions: initial magnetic-TiO_2_-nanocomposite concentration, 0.4 g/L; initial R6G concentration, 10 mg/L. Different small letters indicate significant difference (Duncan’s test, p < 0.05) among treatments (n = 3).
(DOCX)

S6 Fig R6G declining curves of the synthesized magnetic-TiO_2_-nanocomposites without UV-irradiation. (A) pH = 3.0, (B) pH = 7.0, (C) pH = 10.0. Experimental conditions: initial magnetic-TiO2-nanocomposites concentration, 0.4 g/L; initial R6G concentration, 10 mg/L. Different small letters after each line indicate significant difference (Duncan’s test, p < 0.05) among treatments (n = 3).
(DOCX)

S7 Fig Impacts of irradiation wavelength on the R6G photocatalytic degradation of the synthesized magnetic-TiO_2_-nanocomposites. (A) 460 nm, (B) 540 nm. Different small letters after each line indicate significant difference (Duncan’s test, p < 0.05) among treatments (n = 3).
(DOCX)

S8 Fig Percentage of R6G loss during the photocatalysis process. The number in x-axis (0.2, 0.4 and 0.8) indicates the concentration of the synthesized magnetic-TiO_2_-nanocomposites (mg/L), A refers to Fe_x_O_y_/TiO_2_-0.5, B refers to Fe_x_O_y_/TiO_2_-0.35, C refers to Fe_x_O_y_@TiO_2_-0.5, and D refers to Fe_x_O_y_@TiO_2_-0.35, respectively.
(DOCX)

S9 Fig Effects of magnetic-TiO_2_-nanocomposite concentration on R6G photodegradation kinetics. (A) Fe_x_O_y_/TiO_2_-0.5, (B) Fe_x_O_y_/TiO_2_-0.35, (C) Fe_x_O_y_@TiO_2_-0.5 and (D) Fe_x_O_y_@TiO_2_-0.35. Different small letters after each line indicate significant difference (Duncan’s test, p < 0.05) among treatments (n = 3).
(DOCX)
