# Supplementary material for: One-step synthesis of magnetic-TiO2-nanocomposites with high iron oxide-composing ratio for photocatalysis of rhodamine 6G
Source: PLoS One. 2019 Aug 19;14(8):e0221221. doi: 10.1371/journal.pone.0221221 (PMC6699712; doi:10.1371/journal.pone.0221221)
Supplement: S1 Table — (DOCX) [file pone.0221221.s001.docx]

**S1 Table.** Parameters for synthesizing magnetic-TiO_2_-nanocomposites.

| **Name** | **Method** | **Fe^2+^/Fe^3+^**  **(molar ratio)** | **Fe/Ti**  **(molar ratio)** | **Crystallization**  **temperature (°C)** |
| --- | --- | --- | --- | --- |
| **Fe_x_O_y_/TiO_2_-0.5** | One-step | 1:1 | 0.5 | 500 |
| **Fe_x_O_y_/TiO_2_-0.35** | One-step | 1:1 | 0.35 | 500 |
| **Fe_x_O_y_@TiO_2_-0.5** | Two-step | 1:1 | 0.5 | 500 |
| **Fe_x_O_y_@TiO_2_-0.35** | Two-step | 1:1 | 0.35 | 500 |
