# Supplementary material for: One-step synthesis of magnetic-TiO2-nanocomposites with high iron oxide-composing ratio for photocatalysis of rhodamine 6G
Source: PLoS One. 2019 Aug 19;14(8):e0221221. doi: 10.1371/journal.pone.0221221 (PMC6699712; doi:10.1371/journal.pone.0221221)
Supplement: S2 Table — (DOCX) [file pone.0221221.s002.docx]

**S2 Table.** Fe/Ti ratio of synthesizing magnetic-TiO_2_-nanocomposites calculated by EDS results.

| **MNCs** | **Atom percentage (Molecule)** | | | **Fe/Ti ratio** |
| --- | --- | --- | --- | --- |
|  | **O** | **Ti** | **Fe** |  |
| **Fe/TiO_2_-0.35** | 9.10 | 67.83 | 23.07 | 0.34 |
| **Fe/TiO_2_-0.5** | 11.11 | 60.60 | 33.30 | 0.56 |
| **Fe@TiO_2_-0.35** | 10.40 | 65.82 | 23.79 | 0.36 |
| **Fe@TiO_2_-0.5** | 9.89 | 63.99 | 26.13 | 0.41 |
