# Supplementary material for: One-step synthesis of magnetic-TiO2-nanocomposites with high iron oxide-composing ratio for photocatalysis of rhodamine 6G
Source: PLoS One. 2019 Aug 19;14(8):e0221221. doi: 10.1371/journal.pone.0221221 (PMC6699712; doi:10.1371/journal.pone.0221221)
Supplement: S1 Fig — (DOCX) [file pone.0221221.s003.docx]

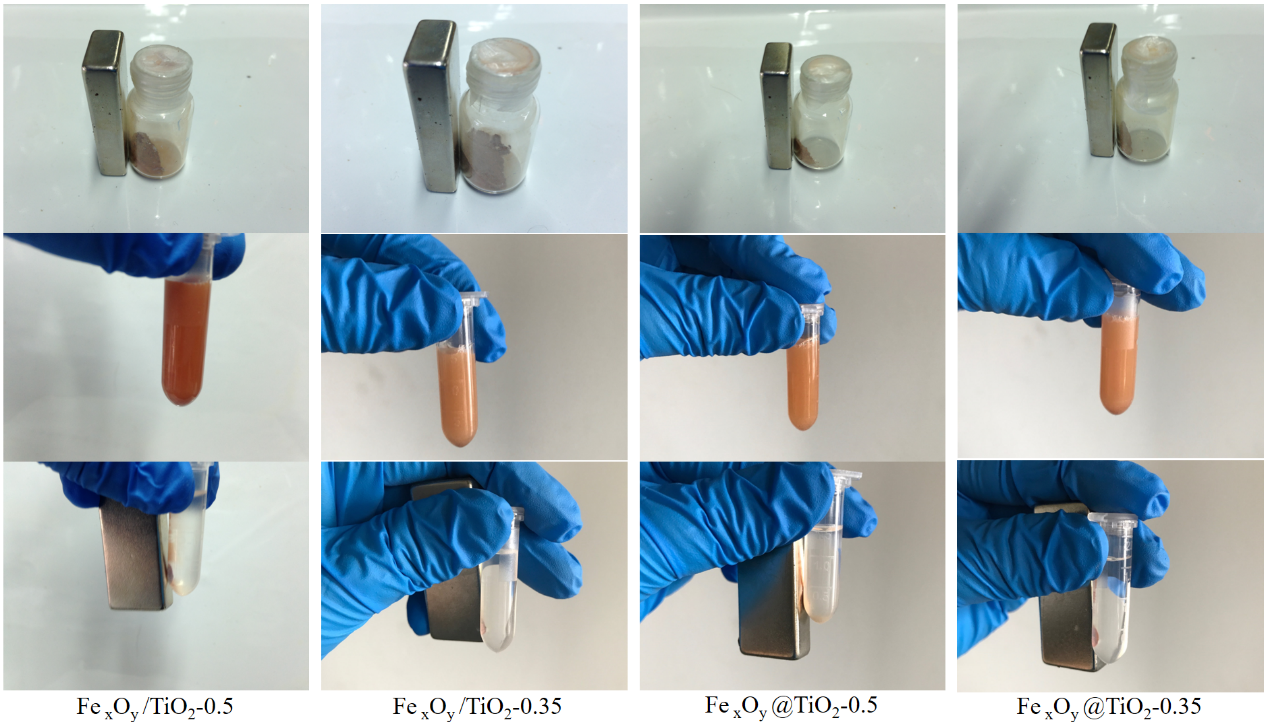


**S1 Fig.** Photographs of synthesizing and harvesting the synthesized magnetic-TiO_2_-nanocomposites.
