# Supplementary figures and images for: One-step synthesis of magnetic-TiO2-nanocomposites with high iron oxide-composing ratio for photocatalysis of rhodamine 6G
Source: PLoS One. 2019 Aug 19;14(8):e0221221. doi: 10.1371/journal.pone.0221221 (PMC6699712; doi:10.1371/journal.pone.0221221)

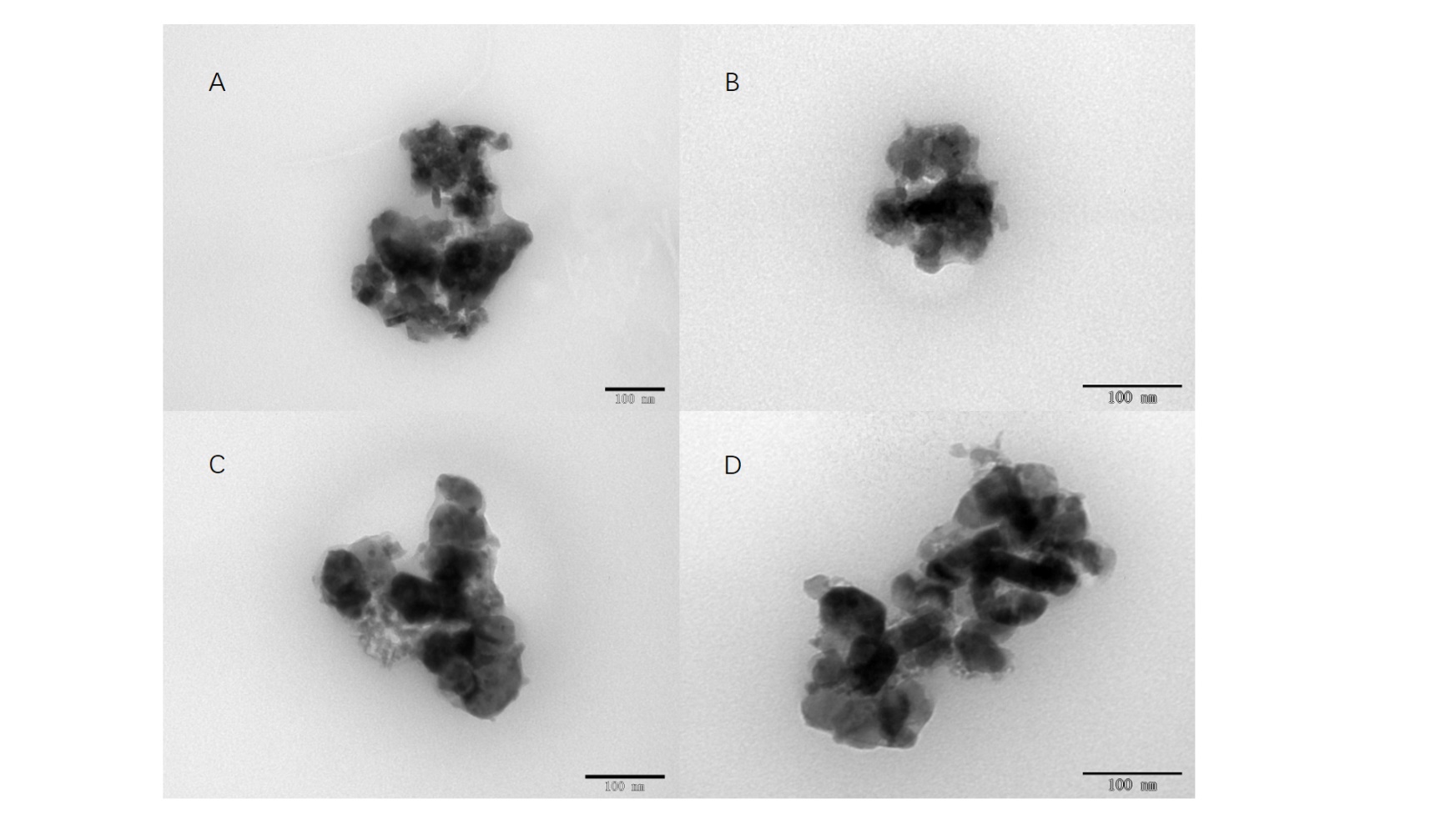


**S2 Fig.** TEM images of (A) FexOy/TiO2-0.5, (B) FexOy/TiO2-0.35 (C) FexOy@TiO2-0.5 and (D) FexOy@TiO2-0.35. Scale bar: 100 nm.

Supplement: S2 Fig — TEM images of (A) FexOy/TiO2-0.5, (B) FexOy/TiO2-0.35 (C) FexOy@TiO2-0.5 and (D) FexOy@TiO2-0.35. Scale bar: 100 nm. (DOCX) [file pone.0221221.s004.docx]
