# Supplementary material for: One-step synthesis of magnetic-TiO2-nanocomposites with high iron oxide-composing ratio for photocatalysis of rhodamine 6G
Source: PLoS One. 2019 Aug 19;14(8):e0221221. doi: 10.1371/journal.pone.0221221 (PMC6699712; doi:10.1371/journal.pone.0221221)
Supplement: S4 Fig — (A) 1 mg/L, (B) 5 mg/L, (C) 10 mg/L, (D) 15 mg/L, (E) 20 mg/L, (F) 25 mg/L. Experimental conditions: magnetic-TiO2-nanocomposites concentration, 0.4 g/L; pH, 7.0. Different small letters after each line indicate significant difference (Duncan’s test, p < 0.05) among treatments (n = 3). (DOCX) [file pone.0221221.s006.docx]

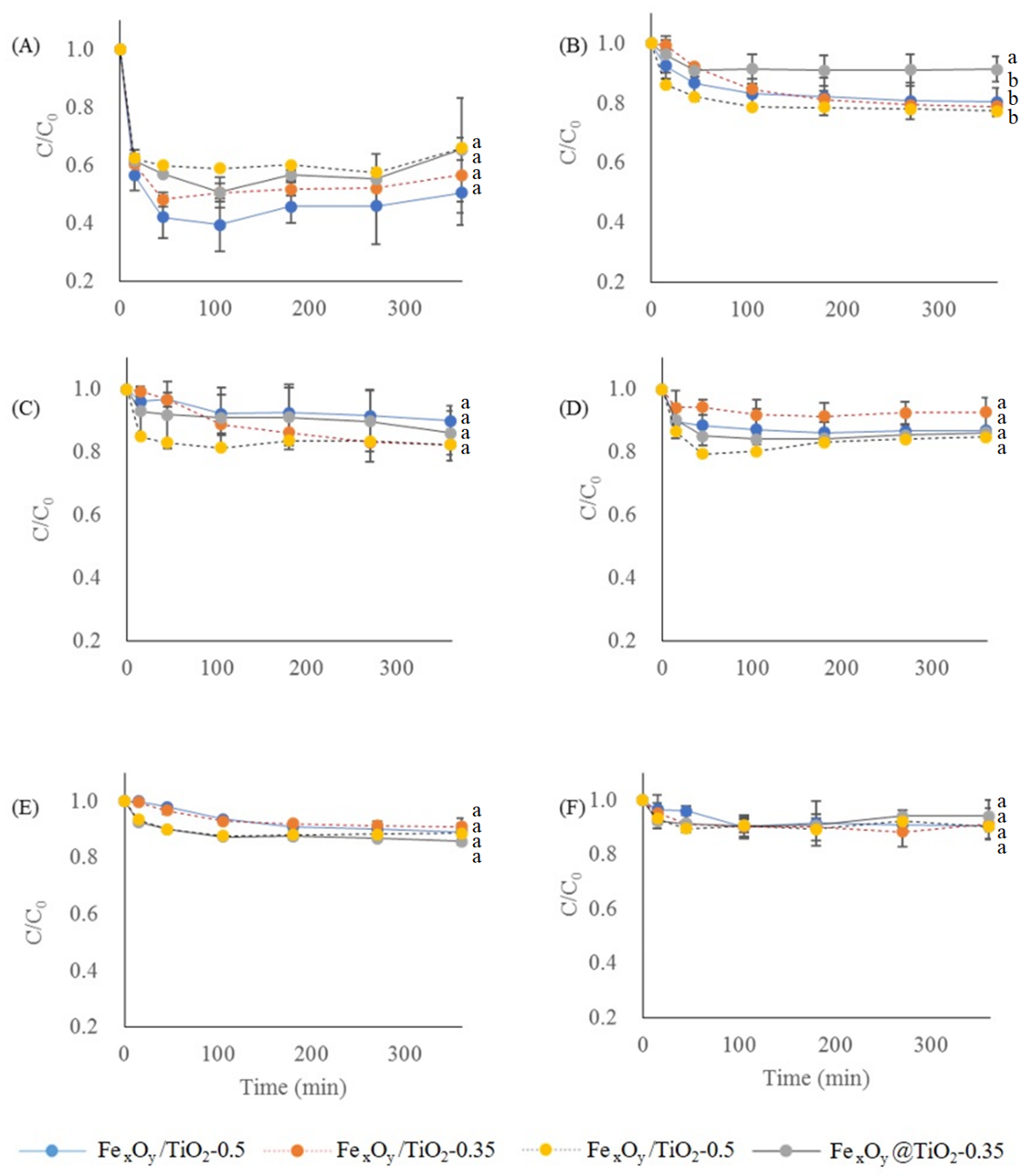


**S4 Fig.** R6G adsorption kinetics on the synthesized magnetic-TiO_2_-nanocomposites. (A) 1 mg/L, (B) 5 mg/L, (C) 10 mg/L, (D) 15 mg/L, (E) 20 mg/L, (F) 25 mg/L. Experimental conditions: magnetic-TiO_2_-nanocomposites concentration, 0.4 g/L; pH, 7.0. Different small letters after each line indicate significant difference (Duncan’s test, p < 0.05) among treatments (n=3).
