# Supplementary material for: One-step synthesis of magnetic-TiO2-nanocomposites with high iron oxide-composing ratio for photocatalysis of rhodamine 6G
Source: PLoS One. 2019 Aug 19;14(8):e0221221. doi: 10.1371/journal.pone.0221221 (PMC6699712; doi:10.1371/journal.pone.0221221)
Supplement: S5 Fig — Experimental conditions: initial magnetic-TiO2-nanocomposite concentration, 0.4 g/L; initial R6G concentration, 10 mg/L. Different small letters indicate significant difference (Duncan’s test, p < 0.05) among treatments (n = 3). (DOCX) [file pone.0221221.s007.docx]

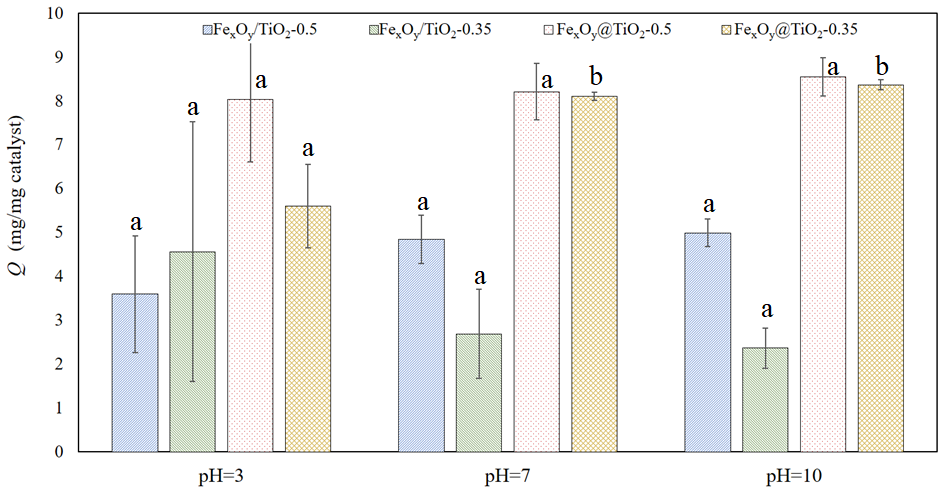


**S5 Fig.** Impacts of pH on R6G adsorption on the synthesized magnetic-TiO_2_-nanocomposites. Experimental conditions: initial magnetic-TiO_2_-nanocomposite concentration, 0.4 g/L; initial R6G concentration, 10 mg/L. Different small letters indicate significant difference (Duncan’s test, p < 0.05) among treatments (n=3).
