# Supplementary material for: One-step synthesis of magnetic-TiO2-nanocomposites with high iron oxide-composing ratio for photocatalysis of rhodamine 6G
Source: PLoS One. 2019 Aug 19;14(8):e0221221. doi: 10.1371/journal.pone.0221221 (PMC6699712; doi:10.1371/journal.pone.0221221)
Supplement: S6 Fig — (A) pH = 3.0, (B) pH = 7.0, (C) pH = 10.0. Experimental conditions: initial magnetic-TiO2-nanocomposites concentration, 0.4 g/L; initial R6G concentration, 10 mg/L. Different small letters after each line indicate significant difference (Duncan’s test, p < 0.05) among treatments (n = 3). (DOCX) [file pone.0221221.s008.docx]

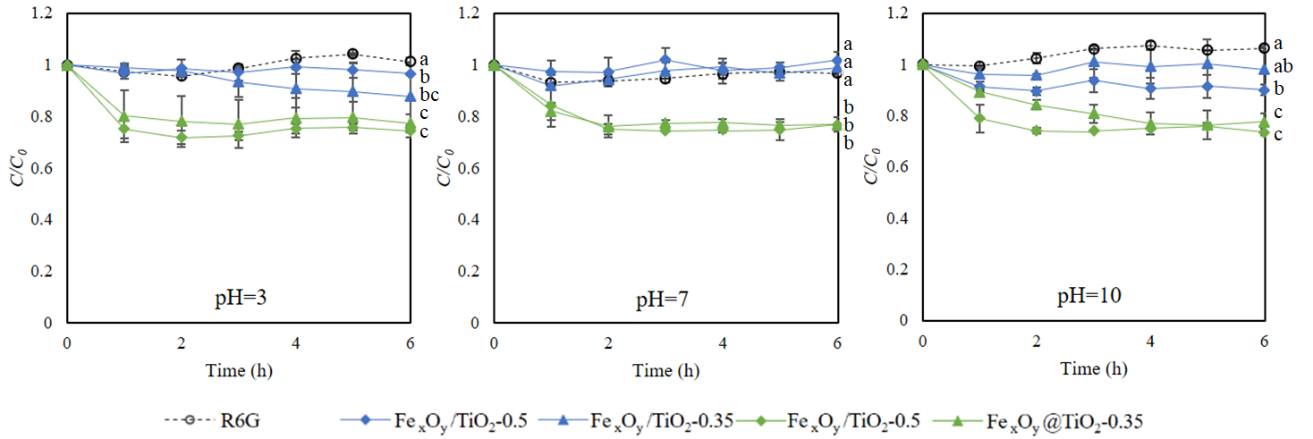


**S6 Fig.** R6G declining curves of the synthesized magnetic-TiO_2_-nanocomposites without UV-irradiation. (A) pH=3.0, (B) pH=7.0, (C) pH=10.0. Experimental conditions: initial magnetic-TiO2-nanocomposites concentration, 0.4 g/L; initial R6G concentration, 10 mg/L. Different small letters after each line indicate significant difference (Duncan’s test, p < 0.05) among treatments (n=3)
