# Supplementary material for: One-step synthesis of magnetic-TiO2-nanocomposites with high iron oxide-composing ratio for photocatalysis of rhodamine 6G
Source: PLoS One. 2019 Aug 19;14(8):e0221221. doi: 10.1371/journal.pone.0221221 (PMC6699712; doi:10.1371/journal.pone.0221221)
Supplement: S7 Fig — (A) 460 nm, (B) 540 nm. Different small letters after each line indicate significant difference (Duncan’s test, p < 0.05) among treatments (n = 3). (DOCX) [file pone.0221221.s009.docx]

**
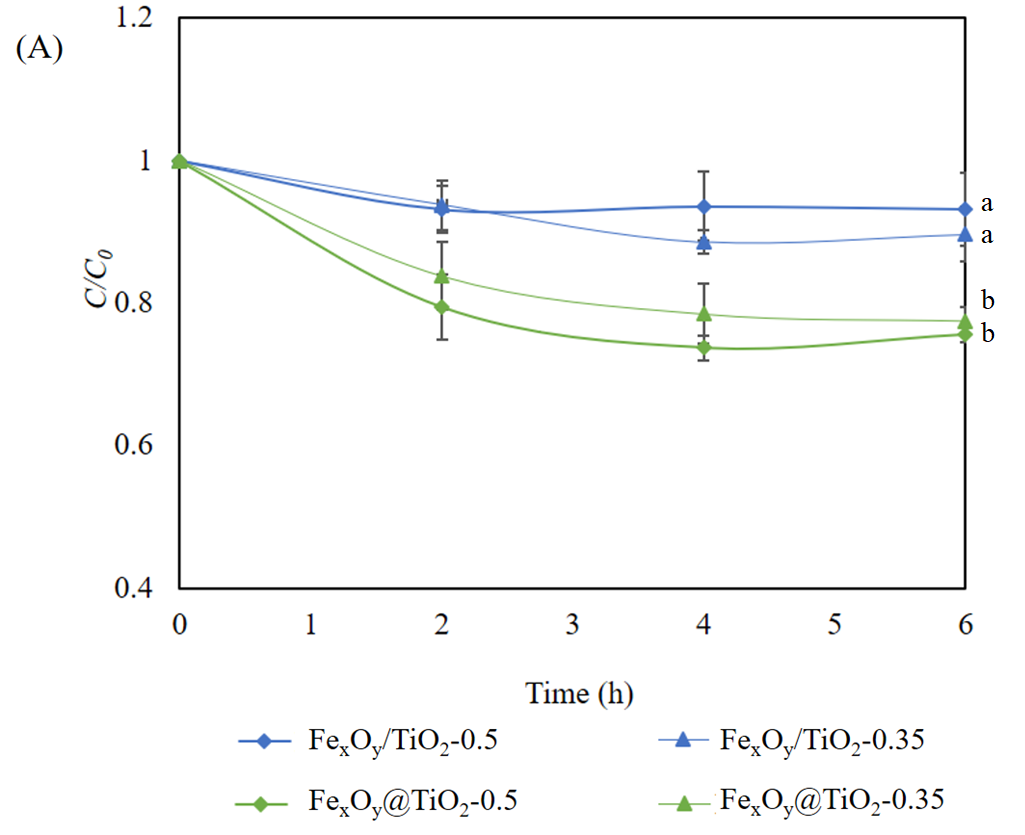
**

**
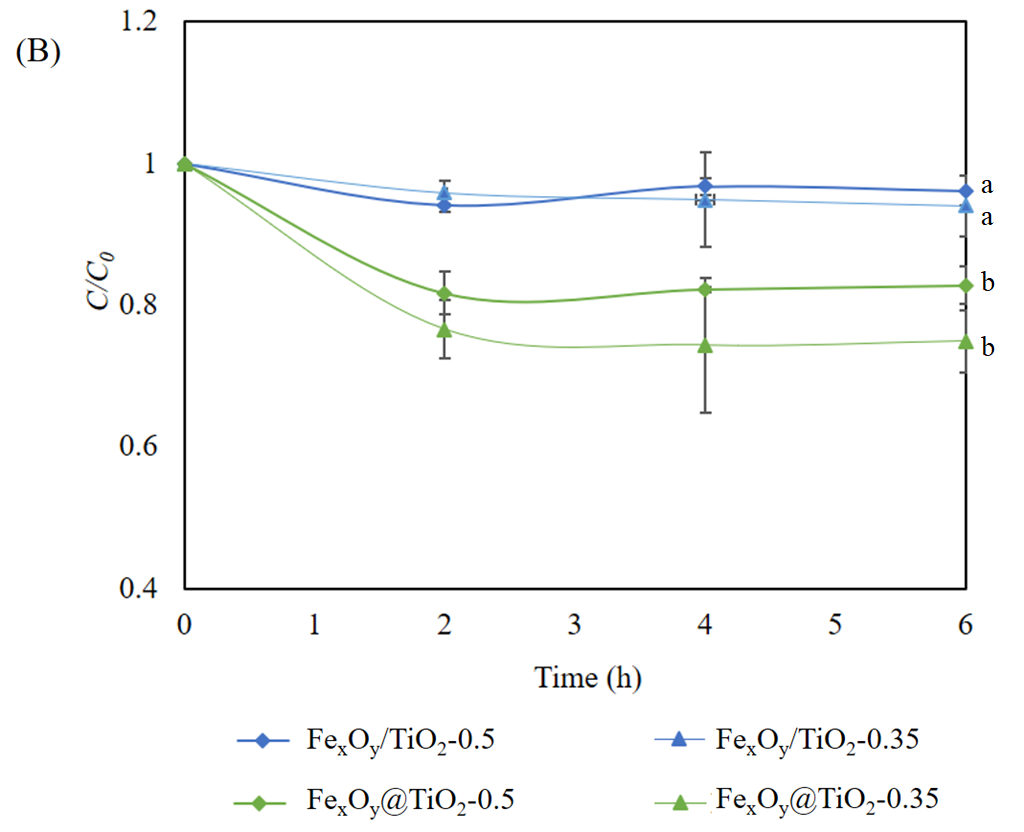
**

**S7 Fig.** Impacts of irradiation wavelength on the R6G photocatalytic degradation of the synthesized magnetic-TiO_2_-nanocomposites. (A) 460 nm, (B) 540 nm. Different small letters after each line indicate significant difference (Duncan’s test, p < 0.05) among treatments (n=3).
