# Supplementary material for: One-step synthesis of magnetic-TiO2-nanocomposites with high iron oxide-composing ratio for photocatalysis of rhodamine 6G
Source: PLoS One. 2019 Aug 19;14(8):e0221221. doi: 10.1371/journal.pone.0221221 (PMC6699712; doi:10.1371/journal.pone.0221221)
Supplement: S8 Fig — The number in x-axis (0.2, 0.4 and 0.8) indicates the concentration of the synthesized magnetic-TiO2-nanocomposites (mg/L), A refers to FexOy/TiO2-0.5, B refers to FexOy/TiO2-0.35, C refers to FexOy@TiO2-0.5, and D refers to FexOy@TiO2-0.35, respectively. (DOCX) [file pone.0221221.s010.docx]

**S8 Fig**. Percentage of R6G loss during the photocatalysis process. The number in x-axis (0.2, 0.4 and 0.8) indicates the concentration of the synthesized magnetic-TiO_2_-nanocomposites (mg/L), A refers to Fe_x_O_y_/TiO_2_-0.5, B refers to Fe_x_O_y_/TiO_2_-0.35, C refers to Fe_x_O_y_@TiO_2_-0.5 and D refers to Fe_x_O_y_@TiO_2_-0.35, respectively.
