# Supplementary material for: One-step synthesis of magnetic-TiO2-nanocomposites with high iron oxide-composing ratio for photocatalysis of rhodamine 6G
Source: PLoS One. 2019 Aug 19;14(8):e0221221. doi: 10.1371/journal.pone.0221221 (PMC6699712; doi:10.1371/journal.pone.0221221)
Supplement: S9 Fig — (A) FexOy/TiO2-0.5, (B) FexOy/TiO2-0.35, (C) FexOy@TiO2-0.5 and (D) FexOy@TiO2-0.35. Different small letters after each line indicate significant difference (Duncan’s test, p < 0.05) among treatments (n = 3). (DOCX) [file pone.0221221.s011.docx]

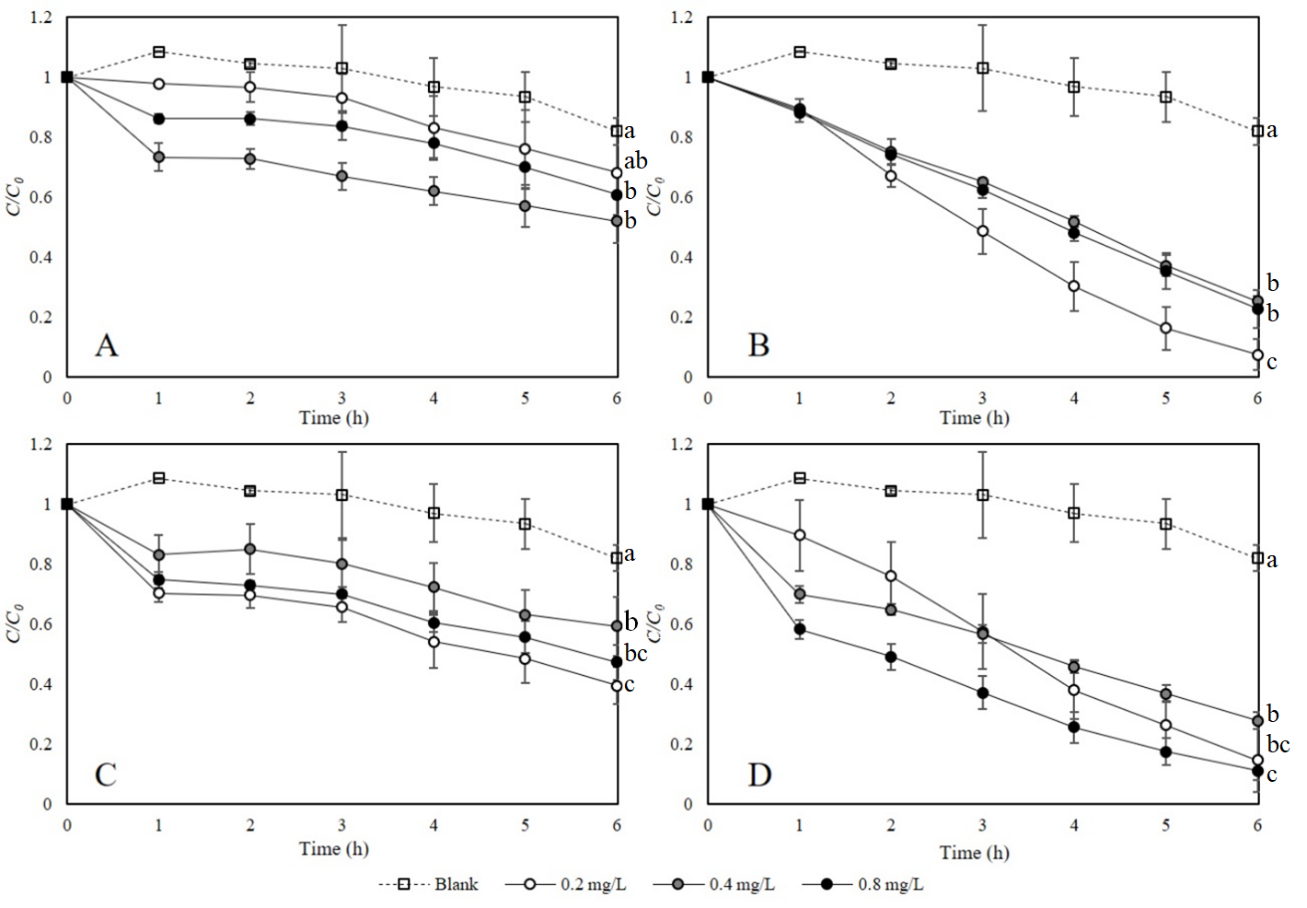


**S9 Fig.** Effects of magnetic-TiO_2_-nanocomposite concentration on R6G photodegradation kinetics. (A) Fe_x_O_y_/TiO_2_-0.5, (B) Fe_x_O_y_/TiO_2_-0.35, (C) Fe_x_O_y_@TiO_2_-0.5 and (D) Fe_x_O_y_@TiO_2_-0.35. Different small letters after each line indicate significant difference (Duncan’s test, p < 0.05) among treatments (n=3).
